# PRISMA: accuracy of response entropy and bispectral index to predict the transition of consciousness during sevoflurane anesthesia: A systematic review and meta-analysis

**DOI:** 10.1097/MD.0000000000025718

**Published:** 2021-04-30

**Authors:** Tao Liang, Fan Wu, Baoguo Wang, Feng Mu

**Affiliations:** a Department of Anesthesiology, Sanbo Brain Hospital, Capital Medical University, Beijing; b Department of Anesthesiology, Affiliated Hospital of Inner Mongolia Medical University, Inner Mongolia, China.

**Keywords:** bispectral index, loss of consciousness, response entropy, recovery of consciousness, sevoflurane

## Abstract

**Background:**

Bispectral index (BIS) and response entropy (RE) are used to monitor the depth of anesthesia.

**Objectives:**

To collect published data and compare the accuracy of BIS and RE in detecting the transition of consciousness during sevoflurane anesthesia.

**Data sources:**

Studies indexed in the PubMed, Embase, or Cochrane databases.

**Study eligibility criteria:**

1. Monitoring of sevoflurane anesthesia depth with BIS and RE simultaneously;
2. Use of prediction probability values to evaluate prediction accuracy; and
3. The full text of the published study is available and contains sufficient data for further analyses.

**Participants:**

Patients who need to use BIS and RE to monitor sevoflurane anesthesia depth simultaneously.

**Interventions:**

A random-effects model was fitted using RevMan 5.3. Subgroup analyses were performed on patient age. The Cochrane *I*^*2*^ methodology was used to determine the heterogeneity of the statistical results, while GRADE Pro served to assess the quality of evidence.

**Results:**

Overall, 195 articles were identified, of which 7 were finally included. The meta-analysis results showed that BIS is more accurate than RE in predicting loss of consciousness (LOC) during sevoflurane anesthesia (MD, .06; 95% confidence interval [CI], .02–.09; *P* = .009; *I*^*2*^ = 92%). In contrast, there was no significant difference between BIS and RE for recovery of consciousness (ROC; MD, .01; 95% CI, .00–.02; *P* = .79; *I*^*2*^ = 83%). Subgroup analyses revealed no significant differences in LOC (MD, .02; 95% CI, .01–.05; *P* = .13; *I*^*2*^ = 60%) and ROC (MD, −.01; 95% CI, −.06–.04; *P* = .58; *I*^*2*^ = 95%) in children. However, the results in adults demonstrated that BIS is more accurate than RE in predicting LOC (MD, −.07; 95% CI, .05–.10; *P* = .002; *I*^*2*^ = 76%).

**Limitations:**

First, this meta-analysis was affected by a large study heterogeneity. Second, this analysis only included publications in English, therefore, some studies may have been omitted.

**Conclusion:**

BIS is more accurate than RE in predicting LOC during sevoflurane anesthesia in adults. However, no significant differences were identified in children.

**Registration number (PROSPERO):**

CRD42020163119

## 1 Introduction

### 1.1 Rationale

Changes in consciousness during anesthesia are a very important aspect related to the mechanisms of anesthesia. The bispectral index (BIS) and response entropy (RE) are both electroencephalography (EEG)-derived methods to monitor the depth of anesthesia.^[1]^ In general, the BIS monitors EEG activity and displays the result as a numeric value between 0 and 100 (high values indicate an alert state, low values deeper levels of hypnosis), while the RE algorithm uses EEG irregularities and frontal electromyogram power spectra, for the calculation. RE values also range between 0 and 100 (inactive to fully alert).^[2]^

The transitions of consciousness during anesthesia comprises 2 parts: anesthetic-induced loss of consciousness (LOC) and recovery of consciousness (ROC). Traditionally, both have been assessed by observing responses to verbal commands.^[3]^ However, the examination method, can strongly influence the results. Although several studies tried to determine the accuracy of BIS and RE in distinguishing the transitions of consciousness, this remains an open question.^[4]^ Addressing this problem may benefit clinical research.

The prediction probability value (PK) has been used in many studies to assess how accurately BIS and RE distinguish consciousness transitions. A PK of .5 represents a 50% success rate to predict the observed state, whereas a PK of 1.0 indicates that this method always predicts the correct state.^[5]^

### 1.2 Objectives

This meta-analysis aimed to collect published data and compare the accuracies of BIS and RE to detect changes in consciousness during sevoflurane anesthesia.

## 2 Methods

### 2.1 Eligibility criteria

This study conforms to the 2009 Preferred Reporting Items for Systematic Reviews and Meta-Analyses (PRISMA) guidelines, and was registered in PROSPERO (CRD42020163119). Because all analyses were based on previously published studies, no ethical approval or patient consent was required. All data were analyzed anonymously during the review process. The methods of analysis and inclusion criteria were specified in advance and documented in the protocol. The inclusion criteria for this meta-analysis were as follows:

1. Simultaneous monitoring of sevoflurane anesthesia depth with BIS and RE;
2. PK use to evaluate prediction accuracy;
3. The full text of the published study was available, and the data sufficient for further analyses.

The exclusion criteria for this meta-analysis were:

1. animal studies and
2. lack of adequate data.

No constraints on language, publication date, or publication status were imposed.

### 2.2 Information sources and search strategies

Two authors (TL and FW) independently searched the following databases for relevant literature: PubMed, Embase, and the Cochrane Library. Systematic reviews published until October 2019 were included. The following keywords were used in the title and abstract fields: “sevoflurane,” “anesthesia, general,” “bis,” “bispectral index,” “RE,” and “entropy.” The electronic search strategy for the 3 databases was defined as follows: ((((“sevoflurane” [MeSH Terms] OR “sevoflurane” [All Fields]) OR “sevoflurane” [All Fields]) OR “sevoflurane s” [All Fields]) AND (“bispectral” [All Fields] OR “bis”[All Fields])) AND ((((“radiation effects” [MeSH Subheading] OR (“radiation” [All Fields] AND “effects” [All Fields])) OR “radiation effects” [All Fields]) OR “re”[All Fields]) OR ((“entropy” [MeSH Terms] OR “entropy” [All Fields]) OR “entropies” [All Fields])).

### 2.3 Study selection and data collection process

The eligibility assessment of the retrieved studies was independently performed in an unblinded standardized manner by 2 reviewers (TL and FW). Disagreements between reviewers were resolved by consensus. The same 2 investigators independently extracted data from publications. A third author (FM) resolved any discrepancies arising during the data extraction and evaluation process. The following information was extracted from each included trial:

1. Trial characteristics including age, sample size, year of publication, and authors;
2. Prediction probability values for RE and BIS;
3. Information regarding the monitor brand; and
4. Methods of consciousness evaluation and reasons for removing data from the present analysis.

### 2.4 Risk of bias

The risk of bias in the included studies was assessed using the risk of bias table in RevMan 5.3. Two authors (TL and FW) answered 7 questions regarding selective reporting, incomplete outcome data, random sequence generation, allocation concealment, blinding of participants, and outcome data to evaluate the risk of bias. The impact of bias on a study was divided into 3 levels: low, high, and unknown risk. For each trial, we plotted the effects against standard errors. The symmetry of the resulting “funnel plot” was visually examined to assess the presence of publication bias in the outcome indicators.

### 2.5 Quality of evidence assessment

Two reviewers (TL and FW) independently evaluated the quality of evidence in accordance with the Grading of Recommendations Assessment, Development, and Evaluation (GRADE) methodology. Assessment items included study design, risk of bias, inconsistency, indirectness, imprecision, and other considerations. The results are divided into different levels (high, medium, low, very low). GRADE Pro was used to construct summary tables for the included studies.

### 2.6 Summary measures and analysis

The primary outcome measure of this analysis was the PK of both BIS and RE to detect changes in consciousness during sevoflurane anesthesia. Data in the current study are presented as mean ± standard deviation. The meta-analysis was performed using RevMan 5.3, with fitting of a random effect model. The Cochrane *I*^*2*^ methodology was used to evaluate the heterogeneity of the statistical results (*I*^*2*^ > 50% and *I*^*2*^ < 25% indicate large and small heterogeneity, respectively). Statistical significance was set at *P* < .05. Subgroup analysis was performed on the patients’ age.

## 3 Results

### 3.1 Study selection

**Figure 1:**
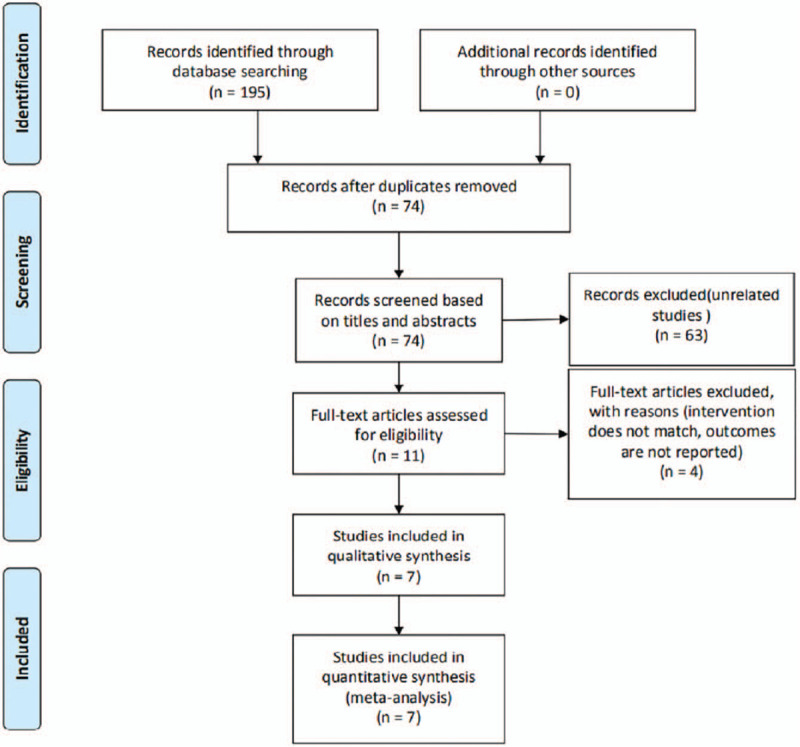
PRISMA flow diagram.

The initial database search rendered 195 articles. After identifying duplicate records, 121 studies were excluded. After reading titles and abstracts, 63 studies were further excluded. After reading the remaining full-text articles in more detail, 7 studies were finally included in this meta-analysis (PubMed 2, Embase 4, and Cochrane Library 1). A flowchart of the publication selection process is shown in Figure 1.

### 3.2 Study characteristics

All 7 studies selected were published in English. A total of 248 samples were included in the selected studies. Five studies collected data from adult patients, whereas 2 (Klockars et al and Sciusco et al) analyzed children data. All studies used the following monitors: BIS (Aspect Medical System, Newton, MA) and RE (GE Healthcare, Helsinki, Finland), except for 1 study which used a different device to monitor BIS (Microsoft Corporation, Redmond, Washington). One study used the observer's assessment of alertness/sedation scale (OAA/s) to evaluate LOC and ROC (Ellerkmann et al), 1 study did not mention the methodology (Klockars et al), and the remaining 5 studies evaluated LOC and ROC by observing patients’ reactions to verbal commands. More information is shown in the overview table, including induction drugs, opioid use, neuromuscular blocker use, type of surgery, and publication date (Table 1).

**Table 1:** Overview of included studies.

ID and author	^1^Richard	^2^Vakkuri	^3^Jaakko	^4^Takamatsu	^5^Kaskinoro	^6^Stefanie	^7^Alberto
Published time	2004	2004	2006	2006	2011	2015	2017
Research type	Observational Cohort study	Observational Cohort study	Observational Cohort study	Observational Cohort study	Observational Cohort study	Observational	
Cohort study	Observational						
Cohort study							
Sample size	32	40	80	20	20	20	36
Age of subjects	21–37 yr	18–65 yr	1–15 yr	22–54 yr	19–30 yr	adults	4–10 yr
Brand information of monitors							
BIS	Aspect Medical, Newton, MA	Aspect Medical, Newton, MA	Aspect Medical, Newton, MA	Aspect Medical, Newton, MA	Aspect Medical Systems	GE Healthcare, Helsinki, Finland	Aspect Medical, Norwood, MA, USA
RE	GE Healthcare, Helsinki, Finland	GE Healthcare, Helsinki, Finland	Datex-Ohmeda Division Corp	GE Healthcare, Helsinki, Finland	GE Datex-Ohmeda S/5TM Entropy Module	Redmond, Washing- ton, USA	GE Healthcare, Helsinki, Finland
Induction anesthetic	Sevoflurane	Sevoflurane	Sevoflurane	Sevoflurane	Sevoflurane	Sevoflurane	Sevoflurane
Opioid use	Not mentioned	Fentanyl	Fentanyl	Not mentioned	None	Remifentanyl	Fentanyl
Neuromuscular blocker use	Not mentioned	Rocuronium	Rocuronium	Succinylcholine vecuronium	None	Succinylcholine	None
Type of surgery	Minor surgery	Elective surgery	Elective surgery	Gynecological surgery	None	Elective surgery	Elective surgery
LOC/ROC evaluation methods	Not mentioned	Modified responsiveness category of the OAA/S to estimate consciousness level	Asking patients to squeeze the investigator's hand	Requesting subjects to open their eyes and observing verbal response	Requesting subjects to open their eyes	Asking patients to squeeze the investigator's hand	Observing continuous purposeful movement, phonation, or eye-opening

### 3.3 Risk of bias

**Figure 2:**
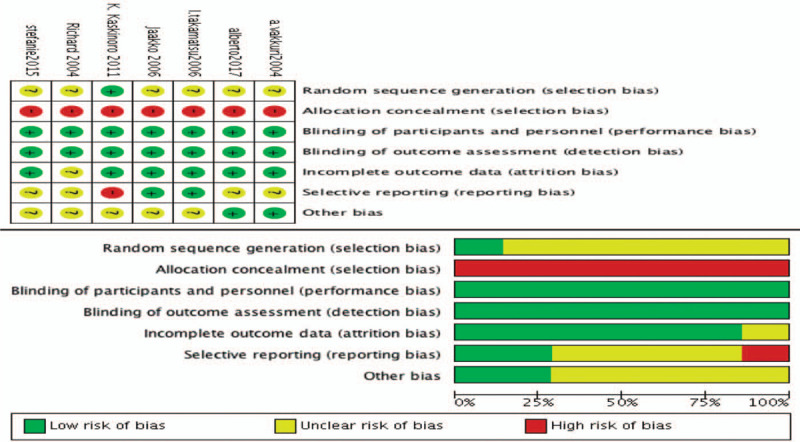
Risk of bias within individual studies.

**Figure 3:**
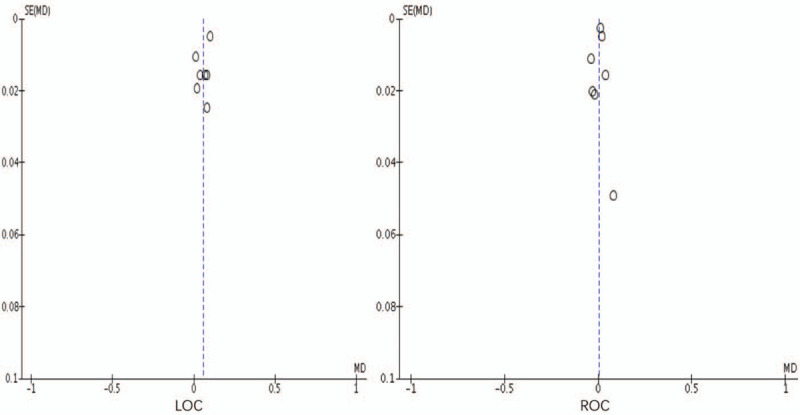
Funnel plot of included studies.

Selection bias was high because allocation concealment and random sequence generation were either not adopted or not mentioned in any studies. The performance, detection, and attrition biases were low. Moreover, reporting bias and other forms of bias were unclear in the majority of included studies. As shown in Figure 2, a strong evidence of large heterogeneity was observed (*I*^*2*^ = 92%), further evidence by the symmetry of the corresponding funnel plot (Fig. 3).

### 3.4 Synthesis of study results

#### 3.4.1 Loss of consciousness

**Figure 4:**
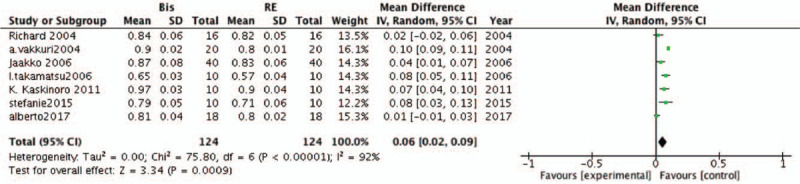
Forest plots of the included studies comparing the accuracy of the bispectral index (BIS) and response entropy (RE) to predict loss of consciousness (LOC).

Seven studies were included in this meta-analysis to estimate the accuracy of BIS and RE in predicting LOC during sevoflurane anesthesia. The results showed that BIS was more accurate than RE in predicting LOC (MD, .06; 95% CI, .02–.09; *P* = .009; *I*^*2*^ = 92%; Fig. 4).

#### 3.4.2 Recovery of consciousness

**Figure 5:**
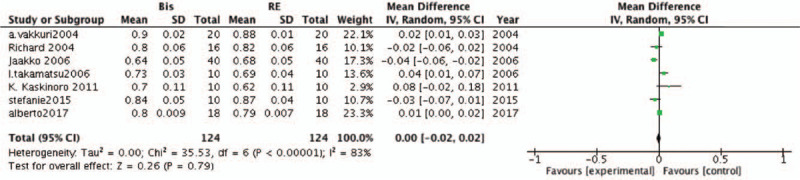
Forest plots of the included studies comparing the accuracy of the bispectral index (BIS) and response entropy (RE) to predict recovery of consciousness (ROC).

In the present meta-analysis, 7 studies were included to estimate the accuracy of BIS and RE in predicting ROC during sevoflurane anesthesia. In contrast to the LOC results, no statistically significant differences were observed between BIS and RE (MD, .01; 95% CI, .00–.02; *P* = .79; *I*^*2*^ = 83%; Fig. 5).

### 3.5 Subgroup analysis for LOC

**Figure 6:**
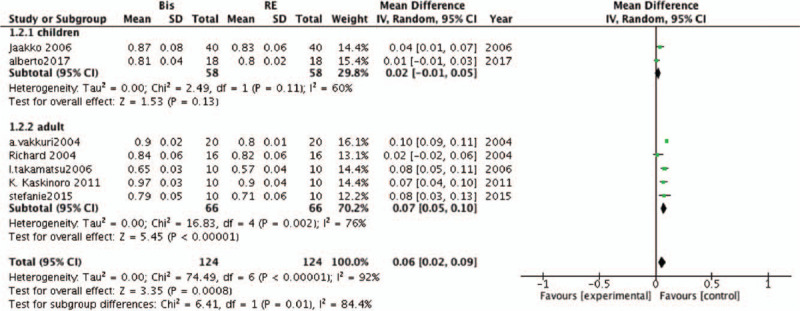
Forest plots of age subgroups comparing the accuracy of the bispectral index (BIS) and response entropy (RE) to predict loss of consciousness (LOC).

Subgroup analyses were performed according to patient age. Five studies included data for adults, whereas 2 studies included children's data. The findings of subgroup analysis indicated that BIS was more accurate than RE in predicting LOC in adults (MD, −.07; 95% CI, .05–.10; *P* < .001; *I*^*2*^ = 76%), but there was no significant difference between BIS and RE in children (MD, .02; 95% CI, −.01–.05; *P* = .13; *I*^*2*^ = 60%; Fig. 6).

### 3.6 Subgroup analysis for ROC

**Figure 7:**
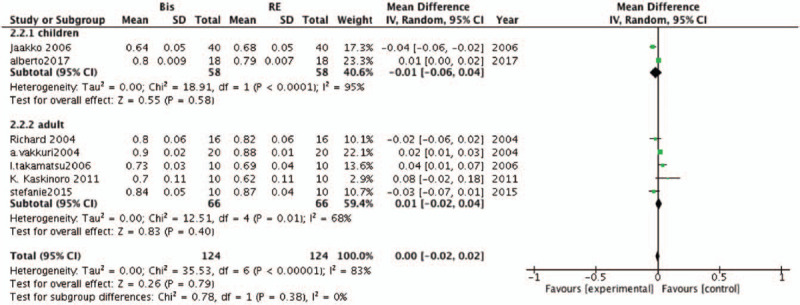
Forest plots of age subgroups comparing the accuracy of the bispectral index (BIS) and response entropy (RE) to predict recovery of consciousness (ROC).

For ROC, the same number of studies were available including adult and children data (5 articles vs 2 articles, respectively). The results of this subgroup analysis suggested no significant differences between BIS and RE either in adults (MD, −.01; 95% CI, −.06–.04; *P* = .58; *I*^*2*^ = 95%) or children (MD, .01; 95% CI, −.02–.04; *P* = .40; *I*^*2*^ = 68%; Fig. 7).

### 3.7 Quality of evidence assessment

**Figure 8:**
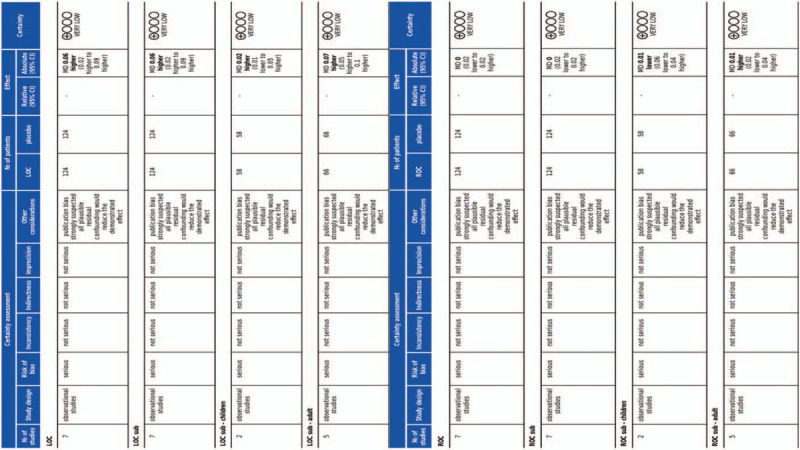
Quality of evidence according to GRADE Pro.

A summary of the quality of evidence according to GRADE Pro is shown in Figure 8. The GRADE levels of evidence for the accuracy of BIS and RE to predict LOC and ROC during sevoflurane anesthesia were very low. Therefore, due to the very low quality of the studies included, we did not perform sensitivity analyses.

## 4 Discussion

### 4.1 Summary of evidence

Many studies have attempted to determine the reliability of the BIS and entropy during sevoflurane anesthesia, while some found that entropy has comparable power to BIS for quantifying the anesthetic drug effect of sevoflurane,^[6]^ this systematic review focusing on transitions of consciousness shows that BIS is more accurate than RE in predicting LOC during sevoflurane anesthesia, even if this finding is affected by heterogeneity. In clinical practice, the entropy monitor has 2 outputs: state entropy and RE. State entropy is calculated in the range of .8 to 32 Hz, predominantly in the EEG, whereas RE is computed from .8 to 47 Hz and includes frontal electromyogram activity.^[7]^ RE is advantageous because it can reveal rapid alterations in frontal cortex activity, while state entropy values are resistant to sudden of facial muscle movements,^[8]^ and this is the reason why RE has been chosen in this meta-analysis as a comparator to BIS in the prediction of consciousness transition during general anesthesia.

Changes in consciousness during anesthesia mainly comprise LOC and ROC. Compared to LOC, exactly determining ROC is more difficult,^[9]^ because the transition from anesthetic-induced hypnosis to consciousness is a complicated phenomenon involving many different neural functions.^[10]^ In clinical anesthesiology, we often judge the state of consciousness by observing the loss and recovery of responsiveness to verbal commands. Due to the subjectivity of this method, results often substantially differ.^[11,12]^ A lack of reaction does not necessarily mean absence of consciousness; some anesthetics may induce an unwillingness to obey commands, while others may impair working memory, so patients immediately forget what they were requested to do.^[13]^ Besides, some drugs induce the suppression of brain motor regions, which can prevent the initiation of movements – another possibility for conscious unresponsiveness.^[14]^ Therefore, the clinical observation of responsiveness is not accurate enough to distinguish consciousness from unconsciousness. Such subjective methods lead to inaccurate results, which may contribute to the heterogeneity among studies.

Sevoflurane is often used to induce general anesthesia. Most studies used 5% sevoflurane inhalation, which may lead to drug effects settling too rapidly for an accurate assessment of RE and BIS values. RE and BIS are computed in specific time windows (1.92 seconds and 15.0 seconds, respectively) which may increase the differences of results.^[15,16]^ Nunes et al point out that compared with BIS, RE resulted in more clinical state misclassifications at the transition from consciousness to unconsciousness.^[17]^ Findings consistent with the results of this meta-analysis.

EEG activity changes with age, therefore this is an important aspect to consider.^[18]^ Two studies included in this analysis included data from children. Although the BIS has been validated in children, its validity in infants is less clear.^[19]^ Some research supports RE's potential to be less influenced by age since it is independent of amplitude and frequency.^[20]^ Klockars et al reported better PKs in children than in infants and also better during induction than emergence. Further, PKs in both age groups were lower than in adults,^[21]^ and most evident in infants. Interestingly, this is also the case for BIS.^[22]^ The study by Davidson et al suggests that BIS and entropy similarly reflect the effect of sevoflurane anesthesia in children, although age has a profound impact on the accuracy of both methods, with better accuracies in increasing age.^[23]^ The results of Ellerkmann et al also confirmed previous evidence that, in infants, BIS and entropy monitors are less linearly correlated.^[24]^ In our meta-analysis, low performance parameters indicate that both BIS and RE fail to capture the state of anesthesia in infants.

### 4.2 Limitations

This meta-analysis had some limitations. First, it was affected by a large study heterogeneity. This may be caused by small sample sizes, differences in protocol design and monitor devices, anesthetic procedures, and surgery types. Second, this analysis only included publications in English; therefore, some studies may have been omitted.

### 4.3 Conclusions

The change in consciousness during anesthesia is an important aspect when studying the mechanisms of anesthesia. The results of this meta-analysis suggest that BIS is more accurate than RE for detecting LOC during sevoflurane anesthesia in adults. Given the heterogeneity of the studies included, caution must be prevailed, especially when extrapolating to infant populations.

## Author contributions

**Data curation:** Feng Mu.

**Formal analysis:** Tao Liang, Fan Wu.

**Funding acquisition:** Feng Mu.

**Methodology:** Feng Mu.

**Project administration:** Fan Wu, Baoguo Wang.

**Resources:** Fan Wu.

**Software:** Tao Liang.

**Supervision:** Baoguo Wang.

**Writing – original draft:** Tao Liang.

**Writing – review & editing:** Baoguo Wang.
